# Fast Pyrolysis of Deashed High-Urea-Formaldehyde Resin Biomass Waste for Platform Chemical and Carbonaceous Fuel

**DOI:** 10.3390/polym18141745

**Published:** 2026-07-16

**Authors:** Xianfang Liao, Haolin Li, Zijie Li, Shuolin Deng, Ronghua Luo, Hang Wang, Qian Yu, Xingwei Yang, Anqing Zheng, Ke Jin, Guoqiang Lv

**Affiliations:** 1 School of Metallurgical and Energy Engineering, Kunming University of Science and Technology, Kunming 650093, China; xianfangliao@outlook.com (X.L.);; 2 State Key Laboratory of Complex Nonferrous Metal Resources Cleaning Utilization in Yunnan Province, Kunming 650093, China; 3 CAS Key Laboratory of Renewable Energy and Guangdong Key Laboratory of New and Renewable Energy Research and Development, Guangzhou Institute of Energy Conversion, Chinese Academy of Sciences, Guangzhou 510640, China; 4 School of Energy Science and Engineering, University of Science and Technology of China, Guangzhou 510640, China; 5 Sinopec Research Institute of Petroleum Processing, Beijing 100083, China

**Keywords:** particleboard sanding powder, urea formaldehyde resin, biomass, deashing pretreatment, fast pyrolysis, pyrolytic char

## Abstract

High ash andsss urea formaldehyde (UF) resin contents in particleboard sanding powder (SP) have restricted the effective resource utilization of SP and make it a hazardous biomass material for particleboard enterprises. To achieve high-value resource utilization of SP while addressing its hazardous disposal issues, different HCl concentration-oriented deashing pretreatments of SP coupled with fast pyrolysis was proposed for producing value-added pyrolytic sugar levoglucosan (LG) and high-quality pyrolytic char. The results show that H^+^ ions released from HCl solution could effectively remove structural ash, likely by disrupting the chemical linkages between the structural ash and lignocellulosic matrix. An amount of 2 mol/L HCl could achieve an over 95% removal rate of alkali and alkaline earth metals (AAEMs) in the ash while maintaining a low loss of polysaccharides. This considerably facilitated the glycosidic cleavage of cellulose into levoglucosan (LG), with the LG yield increasing from 2.18% of raw SP to 13.69% of 2 mol/L HCl deashed SP. Interestingly, it was found that HCl washing of SP facilitated the co-production of value-added platform chemical acetic acid via acid-catalyzed hydrolysis of acetyl groups in UF resin attached to the xylose unit, with the yield increasing from about 7% of raw SP to over 11% of HCl deashed one. Specifically, 2 mol/L HCl deashing pretreatment of SP significantly improved the quality of pyrolytic char with the ash content decreasing from 7.24% to 2.39% and fixed carbon content lifting from 54.08% to 76.04%, thus drastically improving the higher heating value (HHV) from 24.66% of raw SP-derived char to 30.05% of deashed SP-derived char. Moreover, the pyrolytic char CO_2_ gasification reactivity increased from 0.027 min^−1^ of raw SP-derived char to 0.034 min^−1^ of that derived from 2 mol/L HCl deashed SP, approaching that of the widely used industrial charcoal fuel. Pyrolysis kinetic analysis indicates that deashing pretreatment of SP makes the formation of value-added platform chemicals and high-quality carbonaceous fuel proceed more easily at a lower activation energy (214.39 kJ·mol^−1^) than that of raw SP (245.81 kJ·mol^−1^). This study offers a novel approach for the synergistic production of value-added chemicals and high-quality carbonaceous fuel from biomass waste materials with high contents of ash and UF resin, providing a feasible strategy for the clean and high-value resource utilization of wood-based industrial residues.

## 1. Introduction

Particleboard is produced in large quantities and widely applied in producing furniture and upholstery materials in China with the total output exceeding 300 million cubic meters in 2024 [1]. Wood sanding powder (SP), a typical industrial biomass waste characterized by high ash content and high urea formaldehyde (UF) resin content, is generated by sanding machines during the polishing process of particleboard, its annual output in China reaches over 90 million cubic meters. SP features fine particle size, light specific gravity, flammability and explosiveness. Fine particle size and light weight make SP float in the air for a long time, causing severe air pollution and seriously threatening human health. Moreover, the explosiveness characteristics of SP renders it prone to explosion when directly disposed by calcination. SP contains a high nitrogen content (3%~6%) since a large quantity of high-nitrogen binders such as urea formaldehyde (UF) resin is added during its manufacturing process [2], which will be slowly decomposed into formaldehyde and urea, and leads to contamination of soil and water when directly discarded [3]. Notably, conventional direct combustion of SP inevitably emits a large amount of NO_x_ pollutant, causing severe air pollution [4]. Therefore, severe SP pollution issues and difficulty in its resource utilization have become tough problems that have long bothered the Chinese particleboard enterprises.

The main component of SP is lignocellulosic wood material which is chemically composed of polysaccharides (cellulose and hemicellulose) and lignin, in which cellulose is a polymer of glucose linked by *β*-1,4 glycosidic bond and could be converted into sugar-based platform chemical via biochemical or thermochemical routes [5]. On the other hand, SP material contains a large quantity of organic matter with extremely high calorific value, having great potential for preparing high-quality carbonaceous fuel [6]. Synergistic conversion of SP material into sugar-based platform chemical and high-quality carbonaceous solid fuel might serve as a promising route for resource utilization and disposal of SP. Fast pyrolysis, which could convert pretreated lignocellulosic biomass into the highest yield of liquid fuel (>70 wt%) with high selectivity of pyrolytic sugar and could realize co-production of pyrolytic char [7,8,9,10,11,12], is one such a technique that is promising to simultaneously converse pretreated SP into pyrolytic sugar and high-quality carbonaceous fuel. Furthermore, the lignin-derived phenolic compounds in pyrolytic oil can be further upgraded into high-value aromatic fuels and chemicals via catalytic hydrodeoxygenation, which further improves the economic benefits of biomass pyrolysis conversion [13]. However, the fast pyrolytic liquid composition is significantly influenced by ash content of biomass feedstock, especially the alkali and alkaline earth metals (AAEMs). AAEMs in the ash can strongly catalyze the fragmentation of holocellulose to light oxygenates and permanent gases compared to the thermally induced cleavage of glycosidic bonds to form pyrolytic sugar (levoglucosan) [14,15,16,17,18], and even trace levels of AAEMs could intensively diminish the yield of pyrolytic sugar [19]. Moreover, high ash content could also considerably reduce the quality of carbonaceous solid fuel by lowering its calorific value. Dust impurities and metal particles are inevitably introduced during the polishing process of particleboard, which leads to an extremely high ash content of SP (>16 wt%) [20]. Consequently, deashing pretreatment of SP is essential to improve its fast pyrolysis performance for increasing both the selectivity of pyrolytic sugar (LG) and quality of pyrolytic char.

The commonly adopted deashing pretreatment methods include water washing, base leaching, ultrasound radiation, mechanical reduction, air classification, and acid washing [21,22,23]. Ash in biomass is composed of surface and structural ash; the former, surface-soluble inorganic matter (about 20 wt% of the total ash), could be removed by water washing, ultrasound radiation, mechanical reduction and air classification, while the latter is tightly bound in the cross-linking structure of lignin [24] and is extremely difficult to remove by most deashing methods [25]. Base leaching could achieve effective removal of structural ash, while alkali and alkaline earth metal ions such as K^+^, Na^+^, Ca^2+^ will be inevitably introduced and negatively influence the pyrolysis performance, inhibiting the formation of pyrolytic sugar and lowering the caloric value of pyrolytic char. In contrast, HCl washing, which could realize deep removal of bound AAEMs with the ash removal rate reaching over 90 wt% while preserving the integrity of the cellulose framework and improving the caloric value of pyrolytic char [26,27,28], might serve as a promising alternative for deashing pretreatment of biomass feedstock. In contrast, HCl washing can lead to a large mass loss since polysaccharides (i.e., cellulose and hemicellulose) can be partially hydrolyzed, with 15∼20 wt% weight loss of coarse biomass [29].

Acidity and concentration of hydrochloric acid solution are key paramount factors that determine the ash removal rate of biomass and mass loss of polysaccharides, with more ash being removed and more polysaccharides lost as the pH value decreases [29]. Consequently, flexibly regulating the HCl solution concentration so as to strike a balance between the ash removal rate and polysaccharide loss is vital during the deashing pretreatment process of SP. Up to date, in-depth investigations on deashing pretreatment of biomass waste materials such as particleboard sanding powder by different concentrations of HCl solution still remain rarely explored.

**Figure 1:**
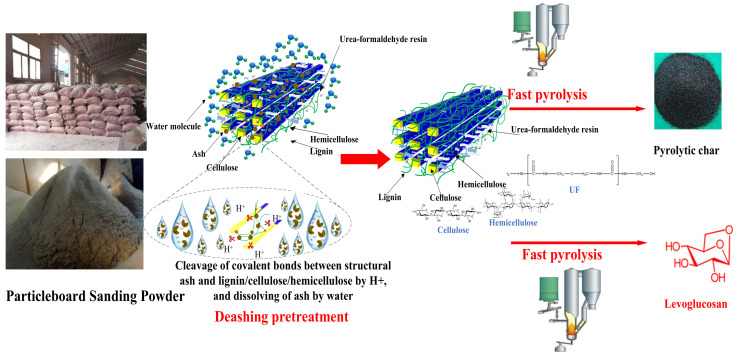
Proposed scheme for pyrolytic sugar and high-quality pyrolytic char production by deashing pretreatment of particleboard sanding powder coupled with fast pyrolysis.

In the current work, deashing pretreatment of SP employing HCl solution at different concentrations for deashing pretreatment of SP was proposed so as to explore the optimal concentration that could achieve high ash removal rate whilst reducing the dehydration induced polysaccharide loss. The deashed SP was subsequently subjected to fast pyrolysis for co-production of pyrolytic sugar (LG) and high-quality pyrolytic char (shown in Figure 1). LG in the pyrolytic liquid can be extracted via a series of mature processes and methods including solvent extraction [30], molecular distillation [31], catalytic hydrogenation-esterification [32], supercritical CO_2_ separation [33] and membrane filtration [34]. LG is a value-added chemical which promises huge market opportunity, and it could be used for producing transportation liquid fuels and chemicals such as ethanol [35], lipid and acid after being firstly converted to glucose by acid hydrolysis [30], or be directly used as significant chiral molecule for the synthesis of antiparasitics, antibiotics and several other drugs [36]. Pyrolytic char with high calorific value, fixed carbon content and gasification reactivity is high-quality carbonaceous fuel which is widely applied as metallurgical fuel [12,37,38] furnace and domestic fuel [39,40,41]. In brief, the very task of this research scheme is to achieve high ash removal of SP while minimizing the polysaccharide loss, thus improving the content of pyrolytic sugar (levoglucosan) in the pyrolytic liquid and obtaining high-quality pyrolytic char during the subsequent fast pyrolysis process. Product distributions and the formation pathway of the target chemicals during the whole process were analyzed in detail. Also, kinetic analysis was conducted to thoroughly analyze the activation energy of deashed feedstock during pyrolysis, providing significant parameters for reactor design and scaling up of particleboard sanding powder conversion into value-added chemicals from laboratory to commercial scale. In brief, the very task of the current work is to improve the concentration of value-added platform chemical (LG) in the pyrolytic liquid and increase the quality of pyrolytic char as carbonaceous fuel via deashing pretreatment integrated with fast pyrolysis.

Specifically, particleboard sanding powder was selected as the research object in this work mainly considering its huge annual output, serious environmental hazards, and representative characteristics of high ash and high UF resin contents, which makes it a typical yet challenging industrial biomass waste for resource utilization. The novelty of this work lies in the systematic investigation of different HCl concentration deashing pretreatment on both pyrolytic sugar yield and pyrolytic char quality, achieving the synergistic co-production of value-added platform chemical (levoglucosan) and high-quality carbonaceous fuel from high-ash- and high-UF-resin-containing biomass waste. This study provides a useful reference for the clean and high-value resource utilization of similar wood-based industrial residues.

## 2. Materials and Methods

### 2.1. Deashing Pretreatment of Feedstocks

Sanding powder (SP), which is derived from the polishing process of wood panels by sanding paper in a furniture mill in Guangzhou, was selected as the feedstock. SP with a particle size of 80~120 mesh was first dewatered in an oven at 105 °C, which was subsequently subjected to water washing (mass ratio of SP to deionized water of 1:200) for 24 h to remove the extractable ash on the surface. The water-washed SP was dried in an oven at 105 °C and kept for further use. Dilute hydrochloric acid solution with different concentrations was employed as the solution for structural ash removal of water-washed SP, and solid and liquid ratio was set as 1:200. Five groups of dilute hydrochloric acid solution at different concentrations were adopted for acid washing, and the specific operational steps for water washing and acid washing are as follows:

(1) Water washing pretreatment: 10 g of dried SP and 2000 g of deionized water were mixed in a 1500 mL glass beaker. The mixture was continuously stirred at 30 r/min using a magnetic stirrer for 24 h. Subsequently, the solid–liquid phases were separated via vacuum filtration. The filter residue was dried in an oven at 105 °C for 24 h, weighed and kept for use in the subsequent acid washing experiments; (2) Acid washing pretreatment: the water-washed SP in the above experiment was used as feedstock and mixed with HCl solutions of 0.1 mol/L, 0.5 mol/L, 1 mol/L, 2 mol/L, and 3 mol/L in a mass ratio of 1:200. The mixture was continuously stirred at 30 r/min using a magnetic stirrer for 24 h; after the acid washing pretreatment came to an end, the solid–liquid phases were separated via vacuum filtration, and the filtered residue was repeatedly washed with deionized water until the solution reached neutral pH. Subsequently, the filter residue was filtered out and dried in an oven at 105 °C for 24 h and kept for use.

### 2.2. Fast Pyrolysis of Raw and Pretreated Feedstock

The fast pyrolysis experiments consist of two parts: for in-depth analysis on the composition and formation pathway of chemicals in pyrolytic oil, one fast pyrolysis experiment is conducted using a pyrolysis–gas chromatograph-mass spectrometer (Py-GCMS), which involves in-depth quantitative and qualitative analysis; in order to collect enough amount of pyrolytic char for further analysis, another batch of fast pyrolysis experiments was performed within a fixed fed pyrolysis reactor, which mainly involves the yield of char, pyrolytic oil and gas, as well as performance testing of the pyrolytic char.

The specific steps for fast pyrolysis and analysis in the pyrolysis–gas chromatography-mass spectrometry (Py-GCMS) system are as follows: (1) 0.2 mg of dried SP sample was weighed and placed into a quartz tube. (2) The quartz tube is inserted into the inlet port of the pyrolysis reactor to ensure a secure seal, followed by the initiation of the pyrolysis program, wherein the resistance wire is rapidly heated to 500 °C by the furnace and maintained at this temperature for 20 s. (3) The split ratio of the autosampler was set to 100:1 and the injector temperature maintained at 250 °C. When the pyrolysis process was completed, the products were transferred from the CDS probe to the gas chromatograph using 99.999% purity helium as the carrier gas. The gas flow rate between the probe and GC column was configured to 20 mL/min, with the GC column gas flow rate set to 1 mL/min; Separation of the products was performed using an Agilent HP-INNO wax capillary column. (4) GC oven temperature program is set as follows: 50 °C held for 2 min, then ramped at 10 °C/min to 90 °C, followed by a 4 °C/min increase to 120 °C, and finally a 8 °C/min rise to 230 °C where it was held for 6.75 min. Quantitative analysis of the yields of the following liquid pyrolysis products was conducted using the NIST mass spectrometry database and the external standard method [8]. Quantitative analysis of the main liquid pyrolysis products (levoglucosan, furan derivatives, C1–C4 oxygenates, phenols, and ketones) was conducted by the external standard method with calibration curves (R^2^ > 0.999). For the remaining compounds, semi-quantitative calculations were performed using structurally similar reference compounds. To ensure the experimental reproducibility and minimize the error, each sample underwent two parallel pyrolysis experiments. The final mass yield of the compound was calculated as the average of the two experimental results. The yield calculation formula for pyrolysis products is as follows:

(1) $$Yield\left(wt\%\right)=\frac{mass\;of\;compound\left(mg\right)}{mass\;of\;sample\left(mg\right)}\times 100$$

Since the quantitative analysis of all the compounds is rather difficult, in order to analyze the variation in N-containing compound in the pyrolytic oil, the yield of N-containing compounds is roughly calculated according to our former work as follows [11]:

(2) $$Yield\;of\;N\;containing\;compound=\frac{specific\;peak\;area}{mass\;of\;sample\left(mg\right)}$$

The specific operational steps for fast pyrolysis of raw and deashed feedstocks in a fed-batch fixed bed reactor are as follows: (1) Tableting of raw and deashed feedstocks: 2 g of feedstocks were accurately weighed and placed into a mold, which was subsequently compressed into a pellet under 9 tons of pressure. This pelletization was adopted to prevent fine powder particles from being entrained by the carrier gas flow and to ensure complete delivery of the sample into the reaction zone. (2) Pre-flowing high-purity nitrogen gas into the pyrolysis apparatus and raise the temperature to the target pyrolysis temperature of 500 °C. This temperature was selected based on our previous studies, which demonstrated that 500 °C is the optimal temperature for fast pyrolysis of biomass to achieve the maximum yield of levoglucosan and other value-added platform chemicals [5]. (3) Once the temperature stabilizes, drop the pelletized sample directly into the center of the quartz tube via the apparatus feed port and maintain the temperature for 10 min (note: since the feedstock input quantity is much larger than in the Py-GCMS experiment and the pelletized sample has a lower effective heating rate due to its compact structure, the holding time was set to 10 min to ensure complete pyrolysis, instead of 20 s in the Py-GCMS experiment). (4) After ceasing heating, continue to flow the carrier gas to prevent sample oxidation. When the furnace was cooled to room temperature, remove the sample, weigh it, and record the results. A Fourier transform infrared (FTIR) spectrometer (TENSOR27, Bruker Optik GmbH, Ettlingen, Germany) was adopted to analyze the structural variations in deashed SP and char from fast pyrolysis of deashed SP.

### 2.3. Detection of AAEMs in Raw and Deashed Feedstock

The content of AAEMs in the raw and deashed feedstocks was determined using an Optima 8000 inductively coupled plasma optical emission spectrometer (ICP-OES, PerkinElmer, Waltham, MA, USA) according to our previous work [9]. The sample preparation procedure is as follows: 0.3 g of dried sample was accurately weighed and recorded. The weighed sample was transferred to a 30 mL digestion glass tube, subsequently approximately 1 mL of extra-pure perchloric acid and 3 mL of nitric acid were added to the digestion glass tube, gently shaking to ensure thorough wetting of the sample. The glass tube was later placed in an LWY84B model far-infrared digestion furnace for digesting at 150 °C for 24 h until complete digestion, leaving approximately 1 mL of liquid in the tube. After the glass tube naturally cooled to room temperature, its inner wall was rinsed 3~4 times with a small volume of deionized water, which was later injected with deionized water until its volume reached 20 mL. Next, 10 mL of the diluted solution was withdrawn from the glass tube and filtered through a 0.22 μm membrane filter and kept in a plastic tube for subsequent analytical steps.

The ICP-OES analysis was performed under optimized operating conditions as described in our previous work [9]. The total and individual contents of AAEMs in the sample are calculated using Formula (3), the percentage of AAEMs relative to the total content is calculated using Formula (4), and the removal rate of AAEMs in the sample is calculated using Formula (5):

(3) $$Content\;of\;AAEMs\left(\frac{mg}{kg}\right)=\frac{mass\;of\;AAEMs\;in\;sample\;\left(mg\right)}{mass\;of\;sample\;\left(kg\right)}$$

(4) $$Percentage\;of\;total\;AAEMs\;\left(wt.\%\right)=\frac{mass\;of\;total\;AAEMs\;in\;sample\;\left(mg\right)}{\begin{array}{c} mass\;of\;total\;AAEMs\;in\\ rawfeedstock\left(mg\right) \end{array}}$$

(5) $$Removal\;rate\;of\;AAEMs\;\left(wt.\%\right)=1-\frac{mass\;of\;total\;AAEMs\;in\;sample\left(mg\right)}{\begin{array}{c} mass\;of\;total\;AAEMs\;in\\ rawfeedstock\left(mg\right) \end{array}}\times 100\%$$

### 2.4. Fourier Transform Infrared Spectroscopy (FTIR) Analysis

In order to reveal the pyrolytic mechanism and formation pathway of specific platform chemicals from fast pyrolysis of raw and deashed SP, FTIR characterization was conducted on 2 mol/L HCl solution-washed SP, and char from pyrolysis of raw and 2 mol/L HCl solution-washed SP using a Fourier transform infrared spectrometer, with the scanning range set to 4000~400 cm^−1^.

### 2.5. Detection of Polysaccharides Lost During HCl Washing Pretreatment

In order to analyze the loss of polysaccharides during the HCl solution washing processes, quantitative analysis of hemicellulosic sugars (xylose, galactose, arabinose, and mannose) and cellulosic sugar (glucose) in the liquid was conducted by high-performance liquid chromatography (HPLC, Waters 2695/Waters 2414, Waters Corporation, Milford, MA, USA). The refractive index detector with its temperature set as 50 °C was adopted for testing, A Hi-Plex Pb column (Agilent Technologies, Santa Clara, CA, USA) with the temperature set at 70 °C used for separation, and deionized water with the flow rate of 0.5 mL/min adopted as mobile phase. Mass yields of hemicellulosic sugar (HS) and glucose were calculated as follows:

(6) $$\begin{array}{c} Hemicellulosic\;sugar\;yield\;(wt\%)\\ \;\;\;\;\;\;=\frac{mass\;of\left(xylose+galactose+\;arabinose+mannose\right)\;in\;filtrate}{mass\;of\;SP}\times 100 \end{array}$$

(7) $$Glucose\;yield\;(wt\%)=\frac{mass\;of\;glucose\;in\;filtrate}{mass\;of\;SP}\times 100$$

### 2.6. SEM Analysis

Scanning electron microscopy (SEM, JEOL JSM-8100U, JEOL Ltd., Akishima, Tokyo, Japan) and energy dispersive spectroscopy (EDS) were employed to obtain the morphological characteristics and elemental distribution information of the pyrolytic char.

### 2.7. Proximate Analysis

Proximate analysis was conducted according to the national standard GB/T 28731-2012 [42] with the adoption of an oven, crucible, and an analytical balance exhibiting a weighing error of ±0.0002 g. All the pyrolysis experiments were duplicated, yielding a final error margin of less than ±0.1%, thereby ensuring the reliability of experimental data.

### 2.8. HHV Analysis

Higher Heating Value (HHV) of biochar is predicted based on the contents of fixed carbon, volatile matter and ash at different pyrolysis temperatures:

(8) $$HHV=0.3536\times FC_{ad}+0.1559\times V_{ad}-0.0078\times A_{sd}$$

### 2.9. Yield of Pyrolytic Char

The yield of pyrolytic char from fast pyrolysis in the fixed bed reactor was determined as follows:

(9) $$Yield\;=\frac{mass\;of\;solid\;product\;after\;pyrolysis}{mass\;of\;solid\;product\;prior\;to\;pyrolysis}\times 100\%$$

### 2.10. CO_2_ Gasification Reactivity of Carbonaceous Reductant

Chemical reactivity, which is also called CO_2_ gasification reactivity, of the pyrolytic char derived from fast pyrolysis of raw and deashed feedstocks was analyzed using a thermogravimetric analyzer (TGA) (Pyris1 TGA, PerkinElmer Instruments, Waltham, Massachusetts, USA), which was compared with the chemical reactivity of forestry charcoal that is widely used as industrial silicon carbonaceous reductant. The specific operational steps are as follows: (1) Pre-purge the TGA with N_2_ (100 mL/min) for half an hour to evacuate residual air from the system. (2) Place 30 ± 3 mg of sample into the crucible. Heat the furnace to 1173 K at a rate of 20 °C/min and maintain at 1173 K for 5 min. (3) Switch from N_2_ (100 mL/min) to pure CO_2_ (100 mL/min) and commence purging for 30 min to protect the coke sample from oxidation during cooling. The moment the purge gas switches from N_2_ to CO_2_ marks the initiation of gasification. (4) Switch off the high-temperature tube furnace and continue purging with N_2_, once the furnace has cooled to room temperature, remove the carbon sample and weigh the post-reaction mass using an electronic balance [10].

(10) $$x=\frac{m_{0}-m_{j}}{m_{0}-m_{\infty }}$$

(11) $$r=\frac{dx}{dt}$$

(12) $$R_{0.5}=\frac{0.5}{\tau_{0.5}}$$

where *m_o_*, *m_j_*, *m*_∞_ denote the weights at initial, time *j*, and final state, respectively. *τ*_0.5_ was the time when the char conversion (*x*) reached 0.5.

### 2.11. Pyrolysis Kinetic Analysis

The activation energy of a reaction is the amount of energy required for a molecule to change from its normal state to an active state, which is prone to the occurrence of chemical reactions; therefore, activation energy is essential for comparing the difficulty degree of the occurrence of the pyrolysis reaction for pretreated biomass feedstocks under different conditions. Distributed activation energy model (DAEM) is widely adopted to describe the relationship between conversion rate and activation energy during biomass feedstock pyrolysis [43]. Since the majority of components in SP is lignocellulosic biomass, the DAEM is adopted for roughly calculating the activation energy during the pyrolysis of raw and deashed SP. The model was firstly put forward by Vand, and later improved and established DAEM mathematical description and theoretical derivation and analysis by Anthony. The model assumes that the whole thermal conversion process is composed of a series of parallel and continuous irreversible first-order chemical reactions. And each chemical reaction has its own activation energy, which is distributed sequentially [44]. The distributed activation energy model is represented by the following equation:

(13) $$\frac{V}{V^{*}}=1-\int_{0}^{\infty }exp\{-k_{0}\int_{0}^{t}exp(-\frac{E}{RT})dt\}f(E)dE\;$$

where V represents the mass loss till time t, %; V^*^ denotes the total mass loss during the whole reaction process, %; k_0_ is the pre-exponential factor, s^−1^; E is the activation energy, KJ/mol; R is the gas constant, 8.314 J/(mol. K); T is absolute temperature, K; f(E) is the distribution of activation energies; and

(14) $$\int_{0}^{\infty }f\left(E\right)=1$$

In the current work, linear non-isothermal heating program was adopted, and temperature changes linearly with time. By substituting the heating rate β=dT/dt into Formula (13), we obtain:

(15) $$\frac{V}{V^{*}}=1-\int_{0}^{\infty }\exp\left\{-\frac{k_{0}}{\beta }\int_{0}^{T}\exp\left(-\frac{E}{RT}\right)dT\right\}f(E)dE$$

where the temperature integral is convergent but cannot be expressed in a finite closed analytical form. Therefore, an approximate analytical solution of the temperature integral was derived using the modified Coats–Redfern method:

(16) $$\int_{0}^{T}\exp\left(-\frac{E}{RT}\right)dT≌\frac{{RT}^{2}}{E}exp(-\frac{E}{RT})$$

Based on the assumption and model above, the DAEM in the current manuscript for pine sawdust pyrolysis could be described by the following formula:

(17) $$\frac{dV}{dt}≅\frac{d\left(∆V\right)}{dt}=k_{0}e^{-\frac{E}{RT}}(∆V^{*}-∆V)$$

where Formula (17) denotes the reaction rate expression of pyrolysis reactions; $∆V^{*}$ is total mass loss within the activation interval of [E, E + ∆V], %; ∆V is the mass loss till time t. %; temperature increases linearly with time; and heating rate β=dT/t. When divided by ∆V on both sides of Formula (17), after further processing, the following formula could be obtained:

(18) $$1-\frac{∆V}{∆V^{*}}=exp\{-\frac{k_{0}}{\beta }\int_{0}^{T}\exp\left(-\frac{E}{RT}\right)dT\}≅exp[-\frac{k_{0}RT^{2}}{\beta E}exp(-\frac{E}{RT})]$$

By applying the Miura–Maki integral approximation method [45] to the DAEM, Equation (18) can be further simplified to:

(19) $$ln\frac{\beta }{T^{2}}=\ln\left(\frac{k_{0}R}{E}\right)+0.6075-\frac{E}{RT}$$

As the same $\frac{∆V}{∆V^{*}}$ under different heating rate β, $\beta /T^{2}$ versus 1/T with −E/R as slope was developed by Formula (19). Therefore, the slope of the linear fitting curve could be adopted for calculating the activation energy E. The values of $\frac{∆V}{∆V^{*}}$ and T were derived from the TG and DTG curves [44].

## 3. Results and Discussion

### 3.1. Analysis of AAEMs in Raw and Deashed Feedstock

In order to investigate the removal of AAEMs from feedstock under different conditions, their respective and total contents in pretreated and un-pretreated samples were displayed in Table 1. The total AAEM content in the SP raw material was as high as 4384.3 mg/kg, exhibiting significant variation in elemental distribution: Ca accounted for the highest proportion, followed by K, Na, and Mg. After 24 h of deionized water washing, the individual and total content of AAEMs in the raw feedstock exhibited substantial reductions, with the removal rates exceeding 50 wt%. Note that after 24 h of acid washing with 0.1 mol·L^−1^ HCl solution, the removal rate of AAEMs was substantially increased to over 80 wt%. And significant enhancement in the removal of AAEMs in SP was achieved by increasing the concentration of dilute hydrochloric acid solution from 0.1 to 3 mol/L, which reached as high as 96.18 wt%.

**Figure 2:**
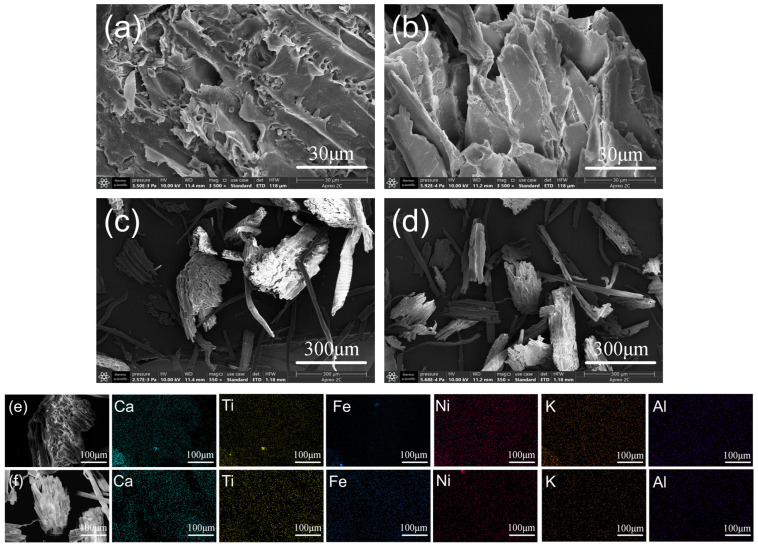
SEM images of raw SP (**a**,**c**) and 2 mol/L HCl-deashed SP (**b**,**d**) before pyrolysis, as well as EDS images of untreated raw SP (**e**) and acid-deashed SP (**f**) prior to pyrolysis.

AAEMs in biomass mainly exist in two forms of ash—namely, extractable ash which could be easily cleared away by physical force, and structural ash located in the cross-linking structure of lignin—which are particularly difficult to remove [8,46]. Over 70 wt% AAEMs exist in the extractable soil ash and less than 30 wt% in the structural ash [47]. To provide direct morphological evidence for the existence of these two ash fractions, SEM-EDS analysis was performed on raw and 2 mol/L HCl-washed SP (Figure 2). The raw SP surface exhibited numerous particulate deposits (Figure 2a,c), which EDS identified as rich in Ca, K, Al, Fe, Ni, and Ti (Figure 2e), corresponding to the surface-adhered extractable ash. After acid washing, these surface particulates were completely removed, exposing a clean, porous fibrous skeleton (Figure 2b,d). Furthermore, EDS elemental mapping revealed that the mineral elements embedded within the fiber matrix were also significantly reduced after acid washing (Figure 2f), confirming that HCl washing can remove both surface extractable ash and internally embedded structural ash. These SEM-EDS observations provide intuitive evidence for the coexistence of two ash fractions in SP.

Table 1 reveals that water washing could primarily remove a majority (55 wt%) of the ash adhered on the surface of SP through physical forces, whereas there was still nearly 15 wt% surface ash that could not be removed by water washing, and little structural ash was removed. Meanwhile, H^+^ ions released from HCl solution could penetrate into the inner structure of SP and bring away the structural ash, simultaneously removing AAEMs. The higher concentration of the HCl solution is, the more H^+^ ions will be dissociated in the solution and more AAEM removal rate could be achieved. Over 94 wt% AAEMs were removed when the concentration of HCl solution reached 0.5 mol·L^−1^, and it is speculated that the yield of LG could be significantly improved.

### 3.2. Analysis of Polysaccharides Lost During HCl Washing Pretreatment

In order to investigate the loss of polysaccharides from HCl solution deashing pretreatment with different concentrations, the yields of hemicellulosic sugars and cellulose derived glucose are displayed in Table 2.

It can be observed that hemicellulose in SP could be hydrolyzed into xylose, galactose, mannose and arabinose, while cellulose hydrolyzed into glucose during HCl solution washing. The yields of these above hydrolyzed sugars tend to increase with an increasing concentration of HCl solution, indicating that lifted HCl solution concentration will result in the loss of polysaccharides in SP. To determine the optimal HCl concentration, a comprehensive evaluation was conducted by integrating multiple indicators including AAEM removal efficiency, polysaccharide loss, and subsequent pyrolysis product performance. Considering that an increased HCl concentration will facilitate AAEM removal (Table 1), the loss of polysaccharides under 1 and 2 mol/L HCl solution is quite similar (≈3 wt%), and significantly lower than that of 3 mol/L HCl solution (>10 wt%); meanwhile, 2 mol/L HCl achieves a higher AAEM removal rate (96.18%) than 1 mol/L HCl, and more importantly, the subsequent pyrolysis results (Section 3.3 and Section 3.4) show that 2 mol/L HCl deashed SP yields the highest levoglucosan production (13.69 wt%) and the best pyrolytic char quality (HHV of 30.05%). Therefore, 2 mol/L is the recommended HCl concentration which could achieve high AAEM removal rate while minimizing the loss of polysaccharides to the lowest extent (<4 wt%), and simultaneously ensures the optimal comprehensive performance of pyrolysis products.

### 3.3. Distribution of Char, Liquid Fuel and Gas from Fast Pyrolysis of Raw and Pretreated SP

**Figure 3:**
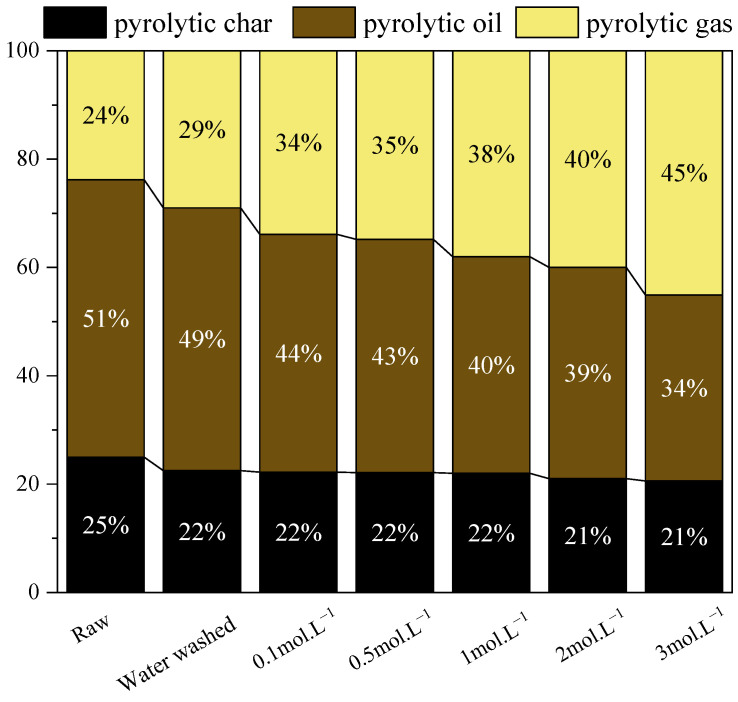
Distribution of pyrolytic char, oil and gas from fast pyrolysis of raw and deashed SP under different conditions.

In order to investigate the effect of AAEMs on the distribution of pyrolytic char, oil and gas, the yield of char, oil and gas from fast pyrolysis of raw and deashed SP feedstock derived from different HCl solution concentrations is displayed in Figure 3.

Obviously, pyrolytic oil comprises the predominant composition with the yield value reaching as high as 51%, followed by pyrolytic char (25%) and gas (24%). After deashing pretreatment by water and HCl solution washing, the yields of pyrolytic oil and char tend to decrease significantly, while that of pyrolytic gas shows an adversely increasing trend. It should be noted that the deashing pretreatment of SP involves two competing effects on pyrolysis product distribution: (1) the catalytic effect of AAEM removal, and (2) the enrichment effect of UF resin. On one hand, the removal of AAEMs by acid washing would theoretically increase the bio-oil yield and decrease the char and gas yields, as it is well established that AAEMs catalyze the formation of pyrolytic char and gas at the expense of bio-oil [48,49,50]. On the other hand, the progressive removal of ash and lignocellulosic components leads to the relative enrichment of UF resin in the deashed SP, which introduces additional nitrogen-containing compounds into the pyrolysis system. The above phenomenon contradicts with the widely reported conclusion that AAEMs facilitate the formation of pyrolytic char and pyrolytic gas while inhibiting the production of pyrolytic oil [49,50,51]. This might be ascribed to the fact that lifted HCl concentration facilitated the removal of ash and impurities from SP and thus leads to drastic enrichment of urea formaldehyde (UF) resin in the deashed UF. Deashed SP with higher content of UF resin contains far higher content of N compounds than the raw SP. Previous studies have demonstrated that UF resin decomposes during pyrolysis and releases various nitrogen-containing gases such as NH_3_, HCN, and HNCO [52], which will be mainly converted into N-containing gas during pyrolysis, thus leading to higher yield of pyrolytic gas with an increasing deashing degree. After the HCl solution concentration reaches 2 mol/L, the yield of pyrolytic char was about 21%, and that of pyrolytic oil reached 39%. Although the yields of char and oil from fast pyrolysis of deashing pretreatment with HCl concentrations higher than 2 mol/L are lower than those from fast pyrolysis of raw SP, it is speculated that the quality of pyrolytic char (heat value) and value-added platform chemicals (levoglucosan) in pyrolytic oil might be significantly improved after deashing pretreatment.

### 3.4. Distribution of Fast Pyrolytic Compounds

**Figure 4:**
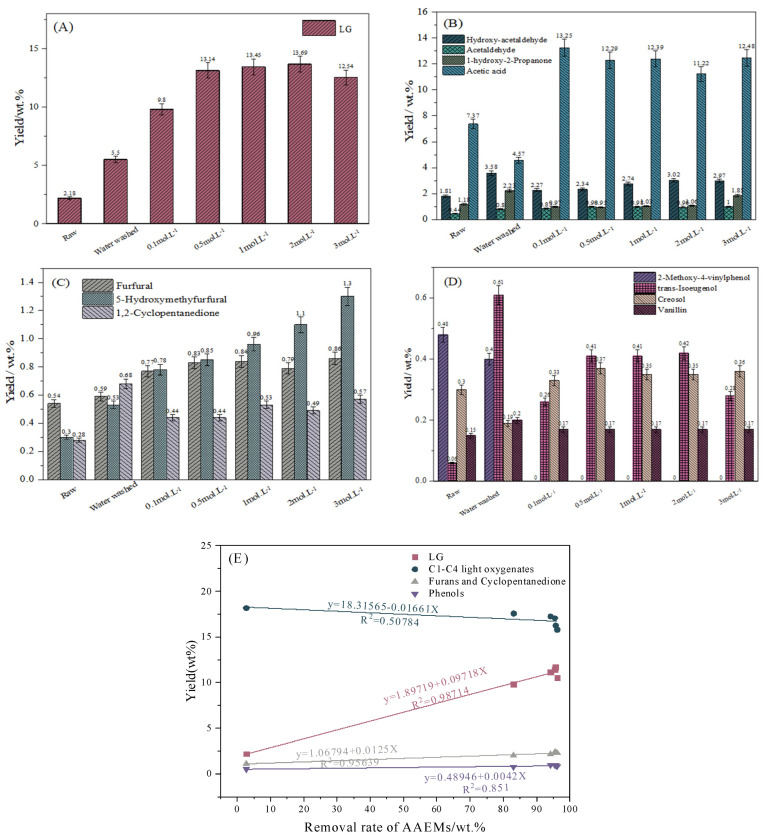
Yield and distribution of liquid pyrolysis products before and after deashing pretreatment ((**A**): levoglucosan; (**B**): C1–C4 light oxygenates; (**C**): Furans and cyclopentanedione; (**D**): Phenols) and yields of liquid pyrolysis products as a function of AAEM removal rate (**E**).

To systematically elucidate how deashing pretreatment reshapes the liquid pyrolysis product distribution, this section analyzes the yield variations in four major product classes—levoglucosan, light oxygenates, furans/cyclopentanones, and phenols—along with the underlying mechanistic drivers. The quantitative and hemi-quantitative analyses of the representative products are shown in Figure 4A–D.

Liquid pyrolysis products from fast pyrolysis of raw and deashed SP mainly include: dehydrated sugars (e.g., levoglucosan), low-molecular-weight C1–C4 oxygen-containing compounds (hydroxy-acetaldehyde, 2,3-butanedione, acetic acid, 1-hydroxy-2-propanone), furans (furfural, 5-hydroxymethylfurfural, 2-methylfuran) and cyclopentanones (1,2-cyclopentanone, 3-methyl-1,2-cyclopentanone), phenols (e.g., trans-isoeugenol, 2-methoxy-4-vinylphenol, methoxyphenol, creosol and vanillin). Figure 4A shows that the yield of the targeted product LG from fast pyrolysis of raw SP was as low as 2.18 wt.%, which is improved significantly to 5.5 wt.% after applying water washing pretreatment, and further improved to 13.69 wt% after applying 2 mol/L HCl solution washing pretreatment. The widely accepted hypothesis for cellulose fast pyrolysis is that it proceeds via two competing routes: the cellulose decomposed either through the cleavage of glycosidic bonds to form LG, or through the AAEM-catalyzed fragmentation of pyranose rings to form low-molecular-weight compounds [53]. Figure 4E shows that the LG yield increases linearly with an increasing AAEM removal rate with the correlation coefficient over 0.98; this coincides well with the hypothesis that the reduced AAEM content could reduce the catalyzed fragmentation of pyranose rings which forms light oxygenates while facilitating the formation of LG via cleavage of glycosidic bonds in cellulose. This strong linear correlation (R^2^ > 0.98) between AAEM removal efficiency and LG yield provides quantitative evidence that AAEM content is the dominant factor governing the competition between cellulose’s glycosidic cleavage pathway and ring-fragmentation pathway, rather than being a secondary influencing factor. Note that there appears no obvious increase in the yield of LG while increasing the HCl solution concentration from 0.5 to 2 mol/L; instead, a decrease in LG yield to 12.54 wt% is observed while increasing the HCl solution concentration from 2 to 3 mol/L. This phenomenon might be related to the fact that more H^+^ ions were left on the deashed SP with an increasing HCl solution concentration, which induces the catalyzed dehydration of cellulose into more furans (furfural and 5-hydroxymethyfurfural) and cyclopentanone [9], thus slightly inhibiting the formation of LG when pretreated with 3 mol/L HCl solution. And this coincides well with the gradually increasing yields of furans (furfural and 5-hydroxymethyfurfural) and cyclopentanone in Figure 4C. Severe hydrolysis of cellulose into glucose and destruction of the cellulose structure might be another reason for the slightly lower yield of LG for 3 mol/L HCl pretreated SP. Figure 4B shows that the yield of C1–C4 low-molecular-weight oxygenates such as acetic acid and hydroxy-acetaldehyde tend to decrease after applying water washing pretreatment, which is mainly attributed to the fact that the removal of AAEMs inhibits the ring-breaking reactions of cellulose to form C1–C4 oxygenates [9]. In contrast, it is noted that the yield of acetic acid turns out to show a drastic increase to over 11 wt% after applying acid solution washing pretreatment. The possible mechanism for the formation pathway of acetic acid will be elaborated in detail in the following part. Figure 4D shows that deashing pretreatment and content of AAEMs do not have an obvious effect on the distribution of phenols during the fast pyrolysis of raw and deashed SP.

In order to analyze the effect of deashing pretreatment on the distribution of N-containing liquid pyrolysis products in the pyrolytic oil, the yield (×10^7^ mg^−1^) of N-containing products from fast pyrolysis of raw and deashed SP under different conditions are presented in Table 3.

As shown in Table 3, six major N-containing compounds were detected in the pyrolytic oil from fast pyrolysis of raw SP by Py-GCMS analysis, with C_5_H_3_F_3_N_2_O_2_S being the predominant one in terms of relative peak area, followed by C_4_H_12_N_2_ and C_3_H_4_N_2_O. It should be noted that these are the main detectable N-containing species above the detection limit; trace amounts of other N-containing compounds may exist but were not identified in the present study. However, after applying water washing and acid solution washing pretreatment, the number of detectable N-containing species were drastically reduced to 2, namely C_2_H_4_N_4_ and C_8_H_17_NO, whose relative yields tend to increase with enhanced removal rate of AAEMs. Overall, two key observations can be summarized regarding the N-containing compound distribution: (1) deashing pretreatment reduces the diversity of small-molecule N-containing compounds in pyrolytic oil; and (2) the remaining long-chain N-containing species show increasing trends with deeper deashing. This demonstrates that the removal of AAEMs in SP could inhibit the formation of short-chain N-containing pollutants during the pyrolysis process. This observed shift from diverse small-molecule N-species to fewer but larger N-containing structures suggests that deashing pretreatment alters the nitrogen transformation pathway: instead of being fragmented into multiple volatile small-molecule pollutants, the nitrogen from UF resin tends to remain in more stable, larger molecular structures that partition preferentially into the char phase.

**Figure 5:**
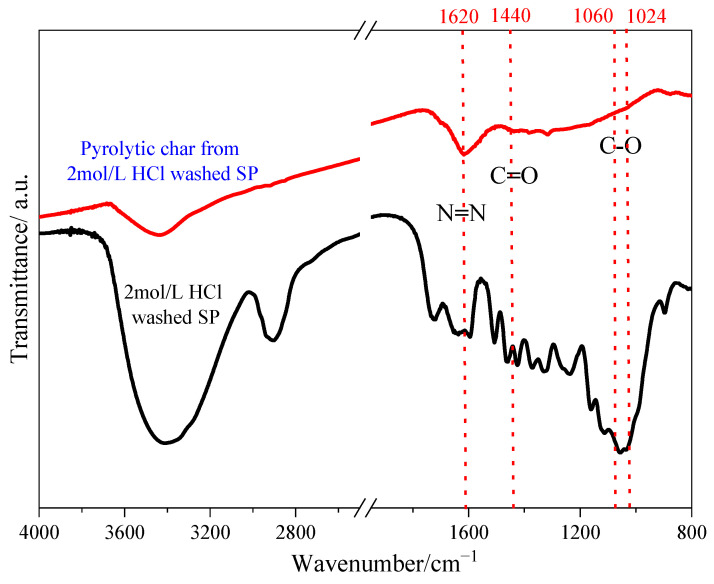
FTIR spectra for 2 mol/L HCl washed SP and solid residue obtained from fast pyrolysis of 2 mol/L HCl washed SP at 500 °C.

In order to reveal the breakage of specific functional groups during the fast pyrolysis of deashed SP and further analyze the formation pathway of specific chemicals in the pyrolytic oil, FTIR spectra for 2 mol/L HCl washed SP and solid residue obtained from fast pyrolysis of 2 mol/L HCl washed SP at 500 °C are depicted in Figure 5. The peaks centered at approximately 1060 and 1024 cm^−1^ are mainly assigned to the C-O vibration of holocellulose [7]. Their peak intensities decreased significantly and even vanished when pyrolyzed at 500 °C, implying that holocellulose within deashed SP decomposed severely after fast pyrolysis at 500 °C. The peak centered at approximately 1440 cm^−1^ is mainly related to the C=O of acetyl groups in UF resin [54], and its peak intensity decreased significantly after pyrolysis at 500 °C, indicating that acetyl groups in UF underwent severe decomposition simultaneously after pyrolysis. The peak at 1620 cm^−1^ resulted from the stretching vibration of the N=N bond, the peak intensity of which decreased slightly, implying that N-containing compound was left in the deashed SP pyrolytic char.

**Figure 6:**
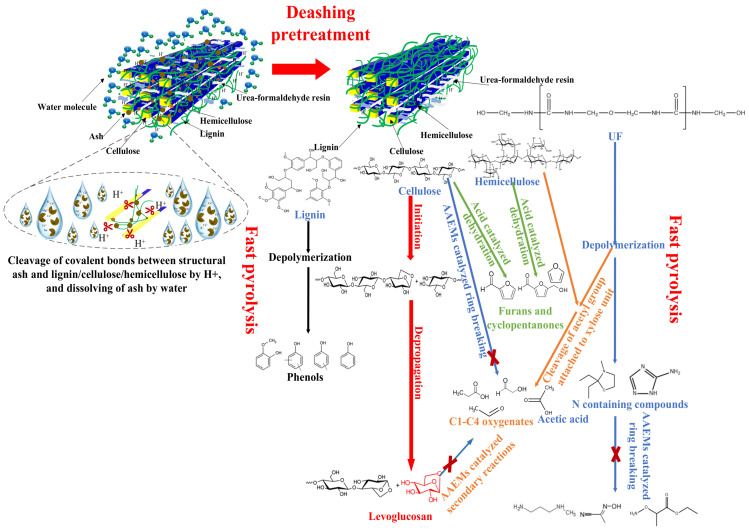
Mechanistic insight into deashing pretreatment of SP coupled with fast pyrolysis for platform chemical production.

For in-depth investigation on the reaction mechanism and formation pathway of platform chemicals during fast pyrolysis of raw and deashed SP, mechanistic insight into the pretreatment and fast pyrolysis process is depicted in Figure 6. For a typical HCl solution deashing pretreatment, feedstock was moistened and expanded by water molecules. Subsequently, hydrogen ions released from hydrochloric acids penetrated into feedstock and played the following roles: breaking the covalent bonds which link the structural ash and lignin/cellulose/hemicellulose. The structural ash was subsequently dissolved and removed in water solution during the stirring process, leading to the removal of AAEMs adhered in the structural ash. The concentration of H^+^ was vital for the deashing of feedstock, the higher concentration of HCl solution will lead to enhanced removal of ash and AAEMs in the feedstock. Lignocellulosic structure with cellulose, hemicellulose and lignin, residual H^+^ ions and minor amount AAEMs as well as large amount of UF (urea-formaldehyde) resin was left in the deashed feedstock.

Based on the product distribution analysis above, the formation pathways of main pyrolytic products can be systematically summarized into five categories (shown in Figure 6), each governed by different controlling factors:

During subsequent fast pyrolysis, liquid pyrolysis products were formed through the following pathways (shown in Figure 5): (1) Macromolecular lignin underwent depolymerization into monophenols. (2) Cellulose underwent initiation and depropagation steps to form levoglucosan through the concerted mechanism [55]: the cellulose chain was firstly broken into a cellulose-like polymer connected with a LG-like end molecule, and a shorter cellulose chain by initial concerted glycosidic cleavage. Subsequently, depropagation steps occurred via breakage of the glycosidic bond to form LG molecules from the chain end of the cellulose-like polymer. The reduced content of AAEMs resulted in an obviously weakened catalytic effect, which significantly facilitated the formation of levoglucosan through initiation and depropagation steps. (3) Light oxygenates (acids, aldehydes and ketones) were mainly formed through thermal-induced and AAEM-catalyzed ring-breaking of cellulose and hemicellulose as well as AAEM-catalyzed secondary reaction of levoglucosan; thus, reduced AAEMs in SP feedstock due to deashing pretreatment contributed to less C1–C4 oxygenates formed. (4) Furans (furfural and 5-hydroxymethyfurfural) and cyclopentanones were mainly formed through acids catalyzed dehydration of cellulose and hemicellulose, enhanced amount of residual H^+^ ions left on the deashed SP feedstock contributed to the increase in the yields of furans and cyclopentanones. Possible reasons accounting for the high yield of acetic acid from fast pyrolysis of deashed SP (extremely high (>11 wt%), see Figure 4B) are as follows: acetic acid is mainly produced during decomposition and catalyzed hydrolysis of acetyl groups (UF resin) attached to the xylose unit under the catalysis of H^+^ ions left from the HCl washing process [52], which is further confirmed by the simultaneous cleavage of C=O bond in acetyl groups and C-O vibration in holocellulose in Figure 5. This demonstrates that co-pyrolysis of UF resin with biomass, especially under H^+^ conditions, facilitates the formation of acetic acid, which coincides well with the result reported in the literature [56]. (5) It could be inferred from Table 3 and Figure 4E that the removal of AAEMs facilitates the depolymerization of long-chain UF into N-containing compounds such as C_8_H_17_NO and C_2_H_4_N_4_, while inhibiting their further AAEM-catalyzed ring-breaking reactions into more low-molecular N-containing pollutants.

In the current research, the highest yield of LG (based on pretreated SP feedstock) reached 13.69 wt.% by 2 mol/L HCl solution washing pretreatment, the corresponding yield of acetic acid was higher than 11 wt.%, and N-containing pollutants were simultaneously reduced in species numbers in pyrolytic oil and left in pyrolytic char. Therefore, 2 mol/L HCl solution washing of SP integrated with fast pyrolysis could be recommended as a preferable process for LG and acetic acid production from the hazardous waste SP feedstock.

### 3.5. Properties of Pyrolytic Char

#### 3.5.1. Proximate Analysis and Heat Value

**Figure 7:**
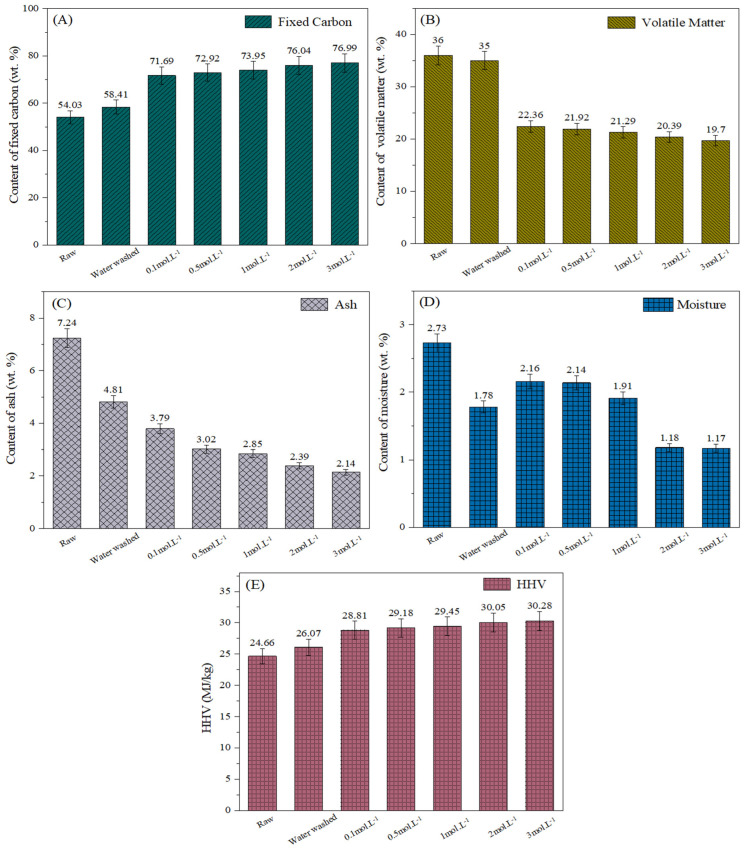
Proximate analysis and high heat value of pyrolytic char ((**A**): fixed carbon; (**B**): volatile matter; (**C**): ash; (**D**): moisture; (**E**) high heat value).

From an industrial application perspective, proximate analysis parameters (fixed carbon, volatile matter, moisture, and ash content) and higher heating value are core quality indicators that directly determine the market value and applicable scenarios of carbonaceous fuels. Figure 7 displays the proximate analysis and high heat value of pyrolytic char under different conditions. As shown in Figure 7, pyrolytic char from fast pyrolysis of the raw SP feedstock contained 54.29% fixed carbon and 36.00% volatile matter with its ash content being as high as 7.24%; After applying deashing pretreatment by water washing, fixed carbon of pyrolytic char increased slightly to 58.82%, whilst volatile matter decreased gradually to 35.00%, and the corresponding ash content decreased drastically to 4.81%. Note that after applying HCl solution washing pretreatment, fixed carbon rapidly increased to 71.69%, which was further improved to nearly 76% with increased HCl solution concentration to over 2 mol/L, and the corresponding ash content was decreased to nearly 2%. As shown in Figure 7E, the high heat value of pyrolytic char was gradually improved with enhanced deashing pretreatment severity and reached as high as over 30 MJ/kg, which is far higher than the heat value (18.23 MJ/kg) of raw SP reported in the literature [57], indicating deashing pretreatment coupled with fast pyrolysis could drastically improve the heat value of SP pyrolytic carbonaceous fuel. Evaluating from the performance of carbonaceous fuel, a high fixed carbon content of 76%, low ash content of 2%, and high heat value of 30.05 MJ/kg for pyrolytic char could be achieved from fast pyrolysis of deashed SP under 2 mol/L HCl solution deashing pretreatment, which could meet the requirement of carbonaceous fuels used in residence heating or industries [58].

#### 3.5.2. SEM Analysis of Char

**Figure 8:**
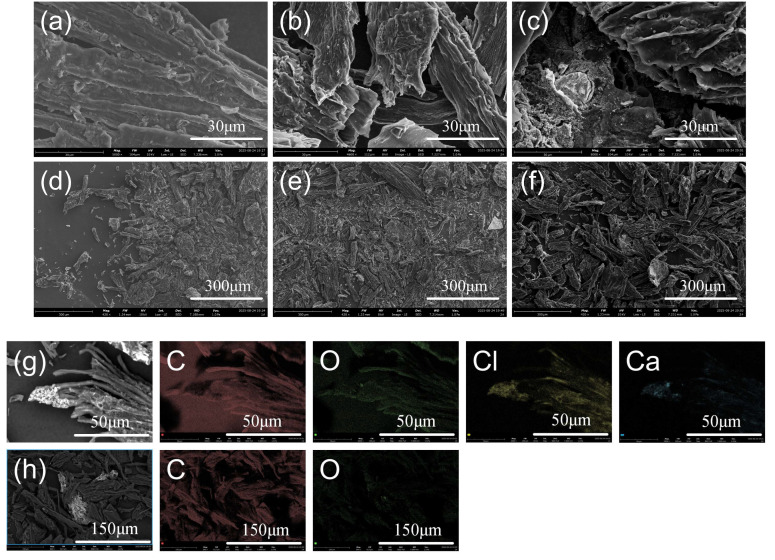
SEM images of pyrolytic char from fast pyrolysis of raw SP (**a**,**d**), water-washed SP (**b**,**e**) and 2 mol/L HCl deashed SP (**c**,**f**), as well as the EDS image of pyrolytic char from fast pyrolysis of raw SP (**g**) and 2 mol/L HCl deashed SP (**h**).

In order to reveal the deashing pretreatment on the morphology of pyrolytic char, SEM characterization of pyrolytic char derived from fast pyrolysis of raw, water-washed and 2 mol/L HCl solution-washed SP was conducted and is displayed in Figure 8.

Figure 8a,d correspond to the SEM images of raw SP pyrolytic char, showing a smooth and surface with undeveloped pore structures. However, the pore structure was obviously enhanced and developed after applying water washing deashing pretreatment, which was further enhanced after applying 2 mol/L HCl solution washing deashing pretreatment. Also, a small number of micropores and mesopores were still observable in HCl solution deashed samples (Figure 8c,f). Although not densely distributed, these pores provide a certain surface area and diffusion pathways for gas–solid reactions, which will to some extent facilitate the gasification rate of pyrolytic char. The development of this porous structure can be attributed to two synergistic effects: (1) the dissolution and removal of inorganic mineral particles embedded in the fiber matrix creates void spaces, and (2) the partial hydrolysis of hemicellulose during acid washing generates additional micro-channels that are further expanded during the subsequent pyrolysis process. EDS elemental analysis (Figure 8g,h) indicates that the signal of Cl was and Ca remarkably reduced after 2 mol/L HCl solution washing deashing pretreatment, indicating that impurities in SP such as alkali earth metals and chlorides could be effectively removed by deashing pretreatment. This coincides well with increased pore structure of pyrolytic char as well as the enhanced removal of AAEMs in Table 1 with an increasing HCl solution concentration.

#### 3.5.3. CO_2_ Gasification Reactivity

**Figure 9:**
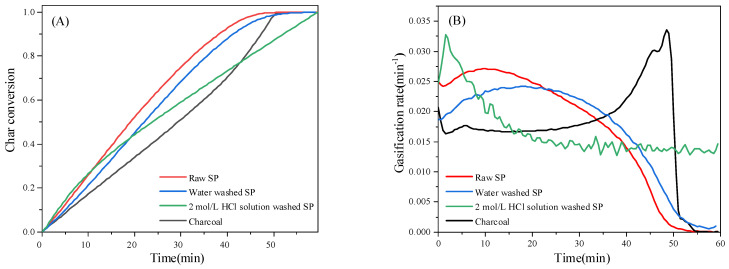
CO_2_ gasification reactivity of pyrolytic chars derived from fast pyrolysis of raw and deashed SP and comparation with that of charcoal. (**A**) Char conversion curves over time; (**B**) Gasification rate curves over time.

As CO_2_ gasification reactivity is also a significant factor for evaluating the performances of carbonaceous fuels used in metallurgical industries, CO_2_ gasification reactivity and gasification parameters of pyrolytic char derived from fast pyrolysis of raw SP feedstock, water-washed SP and 2 mol/L HCl solution-washed SP is depicted in Figure 9 and Table 4. Moreover, CO_2_ gasification reactivity of charcoal, which is widely used as carbonaceous fuel in industries, was also conducted and compared with that of deashed SP pyrolytic char so as to evaluate the performance of deashed SP pyrolytic char.

Figure 9 and Table 4 indicate that the complete gasification time for pyrolytic char derived from fast pyrolysis of raw SP is 45 min, while that for pyrolytic char derived from water-washed SP was prolonged to 49 min, which approaches that of charcoal (50 min). Meanwhile, the complete gasification time for pyrolytic char derived 2 mol/L HCl solution-washed SP is further prolonged to be longer than 60 min, which is mainly related to the removal of AAEMs during deashing pretreatment. It should be noted that the total gasification time reflects the overall conversion kinetics of the char, which is predominantly governed by the catalytic effect of AAEMs throughout the entire gasification process. The removal of AAEMs by acid washing eliminates their catalytic oxygen-transfer cycle effect, thus slowing down the overall conversion rate and extending the complete gasification time [59]. Note that after applying water washing pretreatment, the maximum gasification rate (r_max_) of pyrolytic char was decreased obviously from 0.027 to 0.024 min^−1^, while the maximum gasification rate (r_max_) of pyrolytic char from 2 mol/L HCl solution washing was observed to be increased to 0.034 min^−1^, which is equal to that of the widely adopted carbonaceous fuel charcoal. Similarly, the half-life reaction rate (R_0.5_) of pyrolytic char from fast pyrolysis of 2 mol/L HCl solution-washed SP is 0.021 min^−1^, which is also higher than that of the widely adopted carbonaceous fuel charcoal (0.017 min^−1^). This seemingly contradictory phenomenon—that acid-washed char exhibits a higher maximum gasification rate (r_max_) despite having a longer overall conversion time—can be clarified by distinguishing between initial/peak reactivity and overall conversion kinetics:

(1) Initial/peak reactivity (represented by r_max_): The maximum gasification rate primarily reflects the reaction rate at the early-to-middle stage of gasification, which is strongly influenced by the pore structure and accessible surface area of the char. HCl washing effectively removes inorganic impurities embedded in the carbon matrix, creating a more developed porous structure and larger specific surface area, as confirmed by the SEM images in Figure 8. This enhanced pore structure provides more active sites and facilitates gas diffusion during the initial and middle stages of gasification, leading to a higher peak reaction rate (r_max_). This observation is consistent with previous studies reporting that acid leaching increases the specific surface area of biomass char by extracting inorganic compounds from the biomass matrix, thereby improving the initial gasification reactivity [59].
(2) Overall conversion kinetics (represented by total gasification time): The complete gasification time reflects the overall reaction rate throughout the entire conversion process, which is predominantly determined by the catalytic effect of AAEMs. AAEMs (especially K) can volatilize and mobilize through the char surface during gasification, continuously catalyzing the carbon-CO_2_ reaction via the oxygen-transfer cycle mechanism [59]. As gasification proceeds, the relative concentration of AAEMs in the remaining char increases, further enhancing their catalytic effect in the later stages. In acid-washed char, however, most AAEMs have been removed, so the catalytic effect is absent. Although the improved pore structure accelerates the reaction in the early stage, the lack of catalytic promotion in the middle and later stages leads to a gradual decrease in reaction rate, ultimately resulting in a longer overall gasification time.

Therefore, the higher r_max_ and longer total gasification time of acid-washed char are not contradictory—they reflect different aspects of gasification behavior governed by different controlling factors: pore structure dominates the peak reactivity, while AAEM catalysis dominates the overall conversion kinetics.

Although removal of AAEMs significantly reduces the overall CO_2_ gasification rate of pyrolytic char and extends the complete conversion time, the improved pore structure resulting from removal of impurities could also improve the gasification rate of pyrolytic char at the peak stage, leading to an increase in the maximum gasification rate (r_max_) and half-life reaction rate (R_0.5_) of pyrolytic char.

### 3.6. Pyrolysis Process and Kinetic Analysis

**Figure 10:**
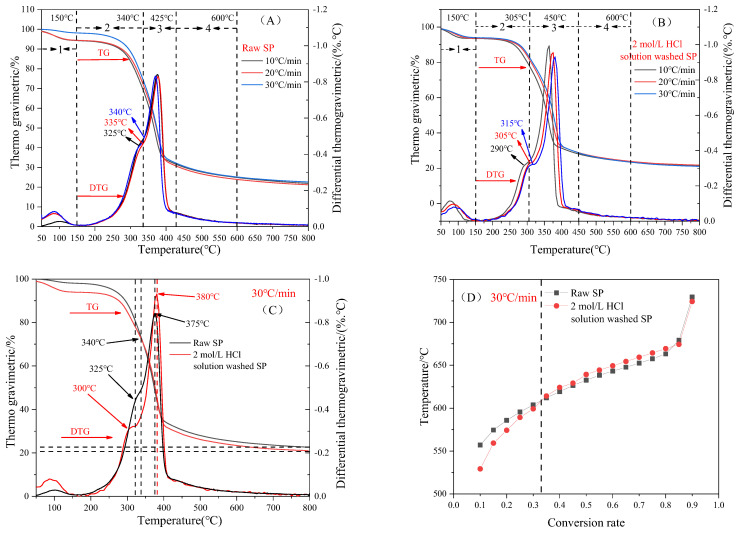
Thermogravimetric curves of raw SP (**A**) and 2 mol/L HCl solution-washed SP (**B**) at different heating rates and the comparison of raw SP and deashed SP at 30 °C⋅min^−1^ (**C**), as well as the corresponding curve of temperature as a function of conversion rate (**D**).

Kinetic analysis provides fundamental parameters for understanding pyrolysis reaction mechanisms and is essential for reactor design, process optimization, and industrial scale-up. Figure 10A,B showed the thermogravimetric curves (TG/DTG) of raw and 2 mol/L HCl solution washing deashed SP under the heating rate of 10, 20 and 30 °C·min^−1^. In order to compare the pyrolysis behavior of raw and 2 mol/L HCl solution washing deashed SP, TG-DTG curves at 30 °C min^−1^ and the corresponding curves of temperature as a function of conversion rate were selected as representative and are depicted in Figure 10C,D. As shown in Figure 10A, pyrolysis of raw SP could be divided into the following four stages: stage 1 (valorization of moisture) occurred from room temperature to 150 °C; stage 2 ranging from 150 °C to the first shoulder peak was mainly ascribed to the valorization of hemicellulose; stage 3, distributed from the first shoulder peak to nearly 425 °C, was the main region for the valorization of cellulose and urea-formaldehyde resin (UF); the following temperature range (stage 4) was the main process for carbonization of lignin and urea-formaldehyde resin (UF). In Figure 10B, the first shoulder peak appeared at a lower temperature (nearly 300 °C) for DTG curves of deashed SP, which might be ascribed to the enrichment of UF after deashing pretreatment, and its pyrolysis temperature shifted towards lower temperature region. As depicted in Figure 10C, from room temperature to 340 °C, the TG curve of the HCl solution-washed, deashed SP moved towards a lower temperature than that of raw SP. This was generally attributed to the fact that UF resin was enriched in the deashed SP after the HCl solution washing pretreatment, the pyrolysis temperature range of UF resin was distributed in a lower temperature range (about 170~450 °C) than lignocellulosic biomass [60], thus contributing to the shift in pyrolysis temperature of the mixture of UF resin and biomass to lower temperature range. However, after 340 °C, the DTG curve of raw SP feedstock tends to shift slightly to a lower-temperature region than that of deashed SP, which is mainly attributed to the fact that the far higher content of AAEMs in raw SP feedstock catalyzed the valorization of three major components of biomass (cellulose, hemicellulose and lignin) to proceed in lower temperature regions. The above analysis agrees well with the change in temperature curves as a function of the conversion rate, as shown in Figure 10D.

**Figure 11:**
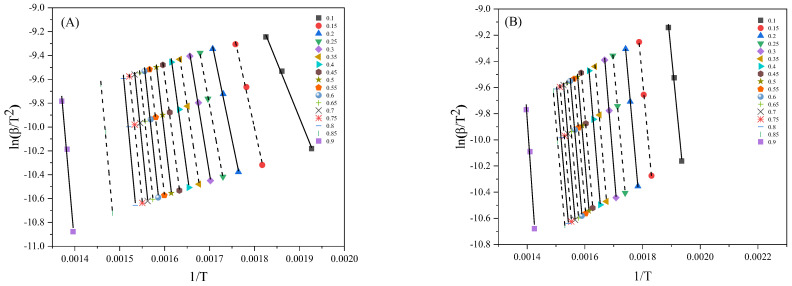
Arrhenius plots of pyrolysis processes for SP feedstock (**A**) and residues from different dilute acid concentrations (**B**) at varying heating rates. Labels: T denotes temperature, β denotes heating rate; each straight line represents a conversion rate (from top to bottom: 30 °C/min, 20 °C/min, 10 °C/min).

**Figure 12:**
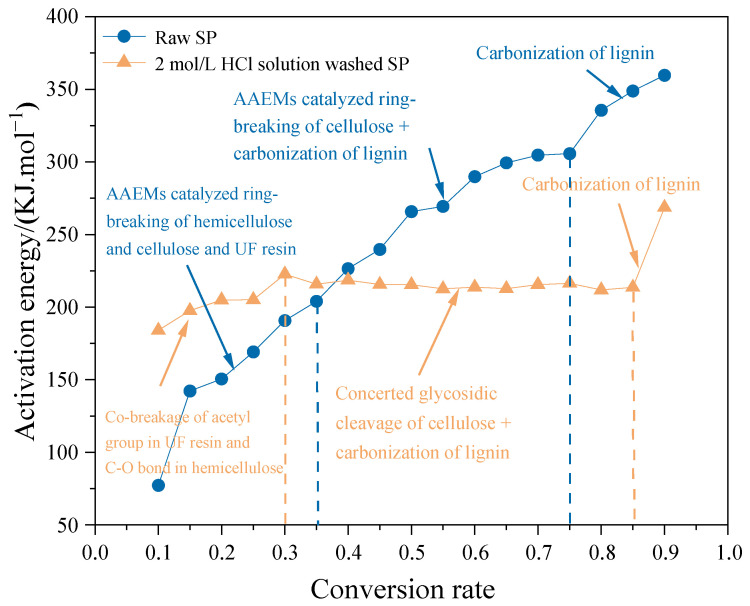
Relationship between the distributed activation energy and conversion rate for fast pyrolysis raw and deashed SP.

Figure 11A,B exhibited the Arrhenius plot of raw SP (A) and deashed SP by 2 mol/L HCl solution washing (B) at different heating rates and selected conversion rates. Figure 12 displays the changes in activation energy with selected conversion rates, and detailed data and information are listed in Table 5. All the kinetic parameters in the current work were calculated based on the temperature range of 150~500 °C. As shown in Figure 11, the vertical coordinate points (*Y* = *ln*(*β*/*T*^2^)) at different heating rates were all linearly fitted to be straight lines. The linear correlation coefficients (*r*^2^) of the fitted lines are all higher than 0.98, demonstrating the DAEM is suitable for calculating the activation energy for the pyrolysis of raw and deashed SP.

Table 5 indicated that activation energy for pyrolysis of raw SP was distributed in the range of 77.19~359.64 kJ·mol^−1^ with the average value of 245.81 kJ·mol^−1^, while that for 2 mol/L HCl solution-washed pretreated SP was slightly decreased to within the range of 184.02~240.58 kJ·mol^−1^ with its average value being 212.74 kJ·mol^−1^.

As shown in Figure 12, for fast pyrolysis of raw SP, the activation energy range (77.19~203.96 kJ·mol^−1^) corresponding to the conversion rate of 0.1~0.35 was regarded as the AAEM-catalyzed ring-breaking of hemicellulose and cellulose into C1–C4 low-molecular-weight oxygenates as well as AAEM-catalyzed ring-breaking of UF resin derived high-molecular-weight N-containing compounds into low-molecular-weight N-containing compounds. The above inferring conclusion is based on the fact that the activation energy range (77.19~190.74 kJ·mol^−1^) is very close to that of hemicellulose and cellulose (94.59 to 208.89), as reported in the literature [9], and a slight decrease in the activation energy to 77.19 kJ·mol^−1^ might be resulted from the lower pyrolysis temperature of UF resin. The following range (conversion rate of 0.35~0.75) for raw SP was the main process for AAEM-catalyzed ring-breaking of cellulose and AAEM-catalyzed carbonization of lignin. The literature reported that cellulose decomposes at nearly 183~199 kJ·mol^−1^ [51], while lignin decomposes at a wider activation energy range of 80~361 kJ·mol^−1^ [51]. Therefore, it is inferred that the conversion rate of 0.35~0.75 corresponding to the activation energy range of 203.96 to 305.66 kJ·mol^−1^ is the comprehensive decomposition process for AAEM-catalyzed ring-breaking of cellulose and carbonization of lignin. And the conversion rate of 0.75~0.9 corresponding to the activation energy range of 305.66 to 359.64 is the main process for carbonization of lignin into pyrolytic char. After the deashing pretreatment, AAEMs were effectively removed, and it is inferred that the pyrolysis process of deashed CF could be divided into three stages: the conversion rate of 0.1~0.3 corresponding to 184.02 to 222.71 kJ·mol^−1^ is the main process for UF resin into acetic acid and H^+^ ions catalyzed hemicellulose into furans and cyclopentanone; the conversion rate of 0.3~0.85 corresponding to 222.71 to 213.60 kJ·mol^−1^ is the main process for concerted glycosidic cleavage of cellulose into levoglucosan as well as carbonization of lignin into char; the conversion rate of 0.85~0.9 corresponding to 213.60 to 268.57 kJ·mol^−1^ is the main region for carbonization of lignin.

Activation energy is the minimum energy required for effective collision reaction of reactants, the lower the activation energy is, the stronger the reaction ability will be. It should be noted that biomass pyrolysis involves multiple overlapping reaction pathways, and the overall activation energy reflects the combined contribution of various parallel and consecutive reactions [61]. After deashing pretreatment by 2 mol/L HCl solution, the average activation energy for pyrolysis of deashed SP is 214.39 kJ·mol^−1^, which is obviously lower than that of raw SP (245.81 kJ·mol^−1^). This value is very close to the characteristic activation energy of cellulose pyrolysis via concerted glycosidic bond cleavage (204.2–212.5 kJ·mol^−1^) reported in previous distributed activation energy model (DAEM) studies [61], indicating that the removal of AAEMs shifts the dominant pyrolysis pathway toward the selective formation of levoglucosan. This demonstrates that the removal of AAEMs in SP by deashing pretreatment makes the concerted glycosidic cleavage of cellulose into levoglucosan and cleavage of acetyl groups in UF resin into acetic acid proceed more easily than pyrolysis of biomass and UF resin to low-molecular-weight oxygenates and N-containing pollutants. Furthermore, the significantly narrower distribution of activation energy for deashed SP (184.02–268.57 kJ·mol^−1^) compared to raw SP (77.19–359.64 kJ·mol^−1^) indicates that deashing pretreatment simplifies the overall pyrolysis reaction network by suppressing the multiple AAEM-catalyzed side reactions, leading to a more selective and uniform thermal decomposition process. This observation is consistent with previous findings that acid washing/deashing pretreatment can effectively enhance levoglucosan production by suppressing the AAEM-catalyzed ring-opening and fragmentation reactions while promoting the transglycosylation pathway [62].

## 4. Conclusions

In the current work, different HCl solution washing pretreatments of particleboard sanding powder with high content of ash and UF resin was performed to strike a balance between the ash removal rate and polysaccharide loss. The deashed SP was subsequently subjected to fast pyrolysis for pyrolytic sugar and carbonaceous fuel production, compared to conventional direct burning; this proposed process significantly reduces the release of N-containing pollutants into the air. The experimental results indicate that H^+^ ions released from HCl solution could effectively remove the structural ash by breaking the covalent bonds linking the structural ash and lignin. By increasing the HCl concentration from 0.1 to 3 mol/L, the AAEM removal rate progressively increased from 83.08% to 96.18%, while polysaccharide loss also rose from less than 1% to over 10%. Among the tested concentrations, 2 mol/L HCl achieved an over 95% removal rate of AAEMs while maintaining a relatively low loss of polysaccharides (<4%), representing a favorable balance between deashing efficiency and feedstock preservation. This considerably facilitated the glycosidic cleavage of cellulose into LG with its yield reaching 13.69%, achieving the synergistic production of acetic acid with the yield reaching over 11%, and simultaneously producing biochar with high heat value (30.05 MJ/kg), and a higher CO_2_ gasification reactivity at low activation energy (214.39 kJ·mol^−1^) than that of raw SP (245.81 kJ·mol^−1^). It should be noted that the deashing pretreatment of SP increased the yield of pyrolytic gas during fast pyrolysis, which may contain nitrogen-containing gaseous species. However, as discussed in Section 3.4, the majority of nitrogen is predominantly retained in the char phase rather than being released as gaseous pollutants, and the diversity of small-molecule N-compounds in pyrolytic oil is also reduced. From a comprehensive perspective considering levoglucosan yield, acetic acid production, char quality, and nitrogen distribution, 2 mol/L HCl represents an optimal pretreatment condition that achieves high-value chemical production and high-quality carbonaceous fuel while minimizing nitrogen-related pollutant emissions compared to direct combustion. This study offers a novel approach for the synergistic production of value-added chemicals and carbonaceous fuel from high N-containing particleboard sanding powder biomass waste feedstocks. Furthermore, the HCl-containing wastewater and ash residues generated during the deashing process can be safely treated via neutralization with lime (Ca(OH)_2_) and solidification, which has been widely adopted in industrial acid-washing wastewater treatment ensuring the environmental sustainability of the proposed process.

## Figures and Tables

**Table 1:** AAEMs content in SP under different acid washing concentrations.

	Raw	Water Washed for 24 h	0.1 mol/L HCl	0.5 mol/L HCl	1.0 mol/L HCl	2.0 mol/L HCl	3.0 mol/L HCl
**AAEMs** **(mg/kg)**			HCl	HCl	HCl	HCl	HCl
**Na**	356.51	157.0	66.20	87.88	87.73	74.37	62.36
**K**	1629.52	68.3	53.87	49.72	46.64	47.01	44.39
**Mg**	305.64	119.7	22.32	17.33	14.63	14.82	12.74
**Ca**	2092.61	1663.0	620.46	107.22	56.21	55.82	52.60
**Total**	4384.30	2008.0	762.9	262.2	205.2	192.0	172.1
**Removal rate**	0.00	55.5	83.08	94.18	95.45	95.74	96.18

**Table 2:** Component analysis of the hemicellulosic and cellulosic sugars in the liquid.

	Water Washed for 24 h	0.1 mol/L HCl	0.5 mol/L HCl	1.0 mol/L HCl	2.0 mol/L HCl	3.0 mol/L HCl
**Yield (wt%)**						
**Hemicellulosic sugars**						
**Xylose**	ND	0.53	1.25	1.78	2.09	3.58
**Galactose**	ND	ND	0.53	0.80	0.86	1.96
**Mannose**	ND	0.21	0.32	0.42	0.55	1.55
**Arabinose**	ND	ND	ND	0.05	0.08	0.29
**Total**	0	0.74	2.1	3.05	3.58	7.38
**Glucose**	ND	ND	0.12	0.29	0.42	2.68

ND: Non-detected.

**Table 3:** Comparison of the yield (×10^7^ mg^−1^) of N-containing compounds identified in fast pyrolysis of raw and deashed SP.

Compounds	Molecular Formula	Deashing Pretreatment Under Different Conditions						
		Raw Material	Water Washing	0.1 mol.L^−1^ HCL	0.5 mol.L^−1^ HCL	1.0 mol.L^−1^ HCL	2.0 mol.L^−1^ HCL	3.0 mol.L^−1^ HCL
3-Amino-striazole	C_2_H_4_N_4_	0.36	0.58	0.74	0.78	0.77	0.82	0.79
Oxazolidine, 2,2-diethyl-3-methyl-	C_8_H_17_NO	0.93	2.16	3.53	3.84	3.84	4.61	4.08
1,3-Propanediamine, N-methyl-	C_4_H_12_N_2_	1.08	0.00	0.00	0.00	0.00	0.00	0.00
Propanenitrile, 2-hydroxy-	C_3_H_4_N_2_O	1.01	0.37	0.00	0.00	0.00	0.00	0.00
Propanoic acid, 2-(aminooxy)-	C_3_H_7_NO_3_	0.46	0.00	0.00	0.00	0.00	0.00	0.00
2,4(1H,3H)-Pyrimidinedione, 5-(trifluoromethyl)-	C_5_H_3_F_3_N_2_O_2_S	3.53	0.00	0.00	0.00	0.00	0.00	0.00

**Table 4:** Gasification parameters of pyrolytic char from fast pyrolysis of raw and deashed SP.

Sample	t_end_ (min)	t_max_ (min)	r_max_ (min^−1^)	R_0.5_ (min^−1^)	Residue (wt%)
Raw	45.0	11.5	0.027	0.026	0.064
Water washed	49.0	18.0	0.024	0.020	0.051
Acid washed	---	1.5	0.034	0.021	0.628
Charcoal	50.0	48.5	0.034	0.017	0.056

**Table 5:** Relationship between pyrolysis activation energy and correlation coefficient for raw and deashed SP at different conversion rates.

Conversion Rate	Raw		2 mol/L HCl Solution-Washed SP	
	Activation Energy/kJ·mol^−1^	R^2^	Activation Energy/kJ·mol^−1^	R^2^
0.1	77.19	0.9977	184.02	0.9954
0.15	142.19	0.9964	197.54	0.9993
0.2	150.52	0.9977	204.86	0.9998
0.25	169.18	0.9989	205.10	0.9997
0.3	190.74	0.9970	222.71	0.9987
0.35	203.96	0.9989	215.77	0.9987
0.4	226.47	0.9925	218.54	0.9981
0.45	239.75	0.9978	215.62	0.9987
0.5	265.76	0.9976	215.38	0.9969
0.55	269.35	0.9976	212.59	0.9986
0.6	289.88	0.9933	213.74	0.9968
0.65	299.35	0.9973	212.76	0.9981
0.7	304.68	0.9976	215.51	0.9985
0.75	305.66	0.9978	216.41	0.9967
0.8	335.58	0.9937	211.86	0.9986
0.85	348.91	0.9948	213.60	0.9979
0.9	359.64	0.9858	268.57	0.9821
Average	245.81	0.9961	214.39	0.9914

## Data Availability

The original contributions presented in this study are included in the article. Further inquiries can be directed to the corresponding author.
